# Clinical Outcomes of Iron Supplement Therapy in Non-Anemic Female CKD Stage 3 Patients with Low Serum Ferritin Level: A Multi-Institutional TriNetX Analysis

**DOI:** 10.3390/jcm14155575

**Published:** 2025-08-07

**Authors:** Hsi-Chih Chen, Min-Tser Liao, Joshua Wang, Kuo-Wang Tsai, Chia-Chao Wu, Kuo-Cheng Lu

**Affiliations:** 1 Division of Nephrology, Department of Medicine, Tri-Service General Hospital, National Defense Medical University, Taipei 11490, Taiwan; shijy630@gmail.com; 2 Department of Pediatrics, Taoyuan Armed Forces General Hospital, Taoyuan City 325, Taiwan; liaoped804h@yahoo.com.tw; 3 Department of Pediatrics, Tri-Service General Hospital, National Defense Medical University, Taipei 114, Taiwan; 4 Department of Research, Taipei Tzu Chi Hospital, Buddhist Tzu Chi Medical Foundation, New Taipei City 23142, Taiwan; j3.reilly@qut.edu.au (J.W.); kwtsai6733@gmail.com (K.-W.T.); 5 Department and Graduate Institute of Microbiology and Immunology, National Defense Medical University, Taipei 114201, Taiwan; 6 Division of Nephrology, Department of Medicine, Taipei Tzu Chi Hospital, Buddhist Tzu Chi Medical Foundation, New Taipei City 23142, Taiwan; 7 Division of Nephrology, Department of Medicine, Fu Jen Catholic University Hospital, School of Medicine, Fu Jen Catholic University, New Taipei City 24352, Taiwan

**Keywords:** iron supplementation, non-anemic, female, CKD stage 3, low ferritin

## Abstract

**Background/Objectives**: Iron deficiency without anemia (IDWA) is common among female patients with chronic kidney disease (CKD), yet the clinical implications of iron therapy in this population remain uncertain. While iron supplementation is frequently used in anemic CKD patients, evidence regarding its outcomes in non-anemic, iron-deficient individuals is limited and conflicting. **Methods**: This retrospective cohort study utilized the multi-institutional TriNetX database to examine the 5-year outcomes of iron therapy in adult women with stage 3 CKD, normal hemoglobin (≥12 g/dL), normal mean corpuscular volume (MCV), and low serum ferritin (<100 ng/mL). Primary outcomes included all-cause mortality, major adverse cardiovascular events (MACE), acute kidney injury (AKI), pneumonia, progression to advanced CKD (estimated glomerular filtration rate ≤30 mL/min/1.73 m^2^), and gastrointestinal (GI) bleeding. **Results**: We identified 53,769 eligible non-anemic patients with stage 3 CKD, low serum ferritin levels, and normal MCV. Propensity score matching (1:1) was conducted on demographic variables to compare iron-treated (*n* = 6638) and untreated (*n* = 6638) cohorts. Over the 5-year follow-up, iron therapy in non-anemic females with stage 3 CKD, low ferritin levels, and iron supplementation was significantly associated with increased risks of MACE, AKI, pneumonia, CKD progression, and GI bleeding (log-rank *p* < 0.0001). No significant difference in all-cause mortality was observed. Data on transferrin saturation and the dosage of iron supplementation were unavailable. **Conclusions**: In non-anemic women with stage 3 CKD and low ferritin levels, iron supplementation was linked to increased MACE, renal, and pneumonia risks without evident survival benefits. These findings suggest that iron therapy in this group of patients may not confer cardiovascular benefit and may pose risks.

## 1. Introduction

Chronic kidney disease (CKD), affecting approximately 10–15% of the adult population, is a significant global health burden. The CKD prevalence among women is higher compared to men, especially in stage 3 CKD [1,2]. Iron deficiency (ID) is a particularly relevant issue in female CKD patients, due to physiological factors such as menstruation, pregnancy, and menopause [3]. Iron deficiency—even in the absence of anemia—is associated with increased risks of fatigue, reduced functional status, cardiovascular disease, and mortality in CKD and kidney transplant recipients [4,5,6,7,8]. More specifically, iron deficiency in CKD patients without anemia has been considered a risk factor for all-cause mortality and CKD progression [9,10,11,12]. Correction with iron supplements is still a dilemma. Current best-practice clinical guidelines for CKD management hypothesize that iron supplementation could be considered for iron-deficient patients without anemia [13]. However, current evidence primarily focuses on patients with anemia, leaving a critical gap in guidance for those with iron deficiency without anemia (IDWA). Clinical judgment and individual patient factors are emphasized in the guidelines, but the absence of clear recommendations for non-anemic patients complicates decision-making, particularly in female CKD patients [14].

A clinically important but often overlooked condition in this context is IDWA—a state characterized by depleted iron stores despite normal hemoglobin levels. IDWA affects up to 30% of non-anemic women with CKD and has been linked to fatigue, reduced exercise tolerance, cognitive impairment, and decreased quality of life—symptoms that are frequently misattributed solely to CKD progression. Moreover, iron plays vital roles beyond erythropoiesis, including mitochondrial function, energy metabolism, and immune regulation, suggesting that untreated IDWA may contribute to adverse clinical outcomes even in the absence of anemia [15].

According to 2019 UNICEF guidelines, iron supplementation is recommended in pregnancy and children but the decision to initiate iron supplementation in non-anemic CKD patients remains controversial [16]. While iron therapy may alleviate symptoms associated with iron deficiency and potentially improve quality of life, it also carries risks, particularly in CKD populations. Excess iron—especially from intravenous administration—has been associated with oxidative stress, endothelial dysfunction, increased infection risk, vascular calcification, and adverse cardiovascular events [17]. Furthermore, concerns exist that iron overload could accelerate renal function decline, posing additional challenges in balancing the benefits and risks of iron therapy [18]. For women with CKD, these concerns are particularly relevant. Women are not only more likely to experience iron deficiency but may also face unique physiological responses to iron supplementation, influenced by hormonal fluctuations and inflammatory diseases such as CKD. Despite this, there is a significant lack of data evaluating the long-term outcomes of iron therapy in non-anemic female CKD patients, leaving clinicians with limited evidence to guide treatment decisions in this population. Given the high prevalence of IDWA among women with CKD and the potential for both clinical benefit and harm with iron supplementation, there is an urgent need for research that specifically addresses this knowledge gap.

This study aims to investigate the long-term outcomes of iron therapy in non-anemic women with stage 3 CKD and low serum ferritin (<100 ng/mL). Utilizing data from the multi-institutional TriNetX network, we evaluate the associations between iron supplementation and key clinical outcomes, including all-cause mortality, major adverse cardiovascular events (MACE), acute kidney injury (AKI), pneumonia, progression to severe CKD (as defined by a glomerular filtration rate (GFR) ≤ 30 mL/min), and GI bleeding. By focusing on this underrepresented patient population, this study seeks to inform evidence-based, sex-specific treatment strategies and optimize iron management in female CKD patient care.

## 2. Materials and Methods

### 2.1. Study Design

This retrospective cohort study utilized the TriNetX network and adhered to the Strengthening the Reporting of Observational Studies in Epidemiology (STROBE) guidelines. TriNetX, a global network of electronic health records from healthcare organizations, facilitates the querying of deidentified patient data. The de-identification process has been verified to comply with Section §164.514 (b) (1) of the HIPAA Privacy Rule. Data from the Global Collaborative Network, which includes 147 healthcare organizations, were queried using ICD-10 codes that aligned with the study’s inclusion and exclusion criteria. Data collection was completed on 31 January 2020. This study protocol was reviewed and approved by the Taipei Tzu Chi Hospital Institutional Review Board, approval number 14-IRB027 (10 March 2025).

### 2.2. Study Cohorts

**Figure 1:**
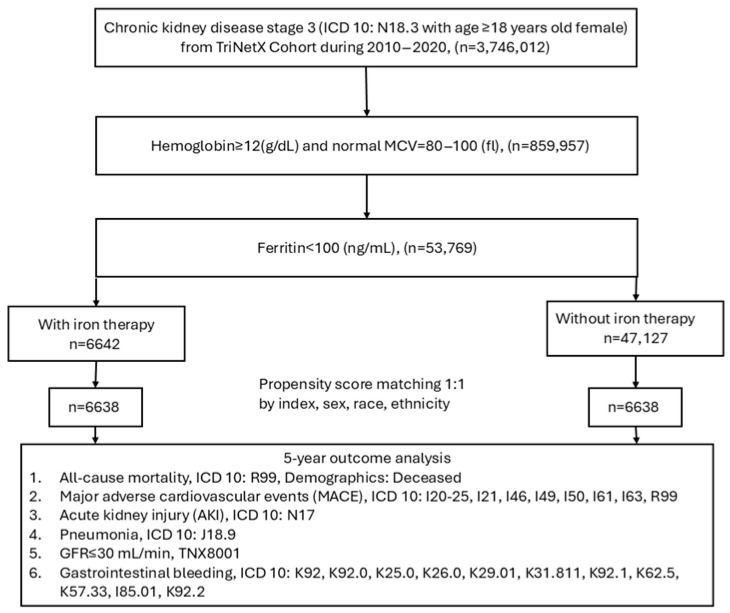
Algorithm for patient selection and enrollment in the study.

The structured flowchart detailing the patient selection criteria, cohort characteristics, and analytical methodology for assessing the long-term (5-year) outcomes of iron therapy in females with CKD stage 3 can be seen in Figure 1. The study includes 3,746,012 individuals aged 18 years and over, diagnosed with moderate CKD (ICD-10: N18.3) between 1 January 2010 and 31 January 2020. Only patients with normal hemoglobin (>12 g/dL) and RBC mean corpuscular volume (MCV: 80–100 fL), and serum ferritin levels below 100 ng/mL (*n* = 53,769) at the time of diagnosis were included. Anyone who developed anemia (hemoglobin < 12 g/dL) during the follow-up period was not included in this study. The study compares patients receiving iron therapy (≥1 documented instance within 3 months before or up to 3 years after the diagnosis of stage 3 CKD with normal hemoglobin and MCV, *n* = 6642) with those not receiving it (*n* = 47,127), using 1:1 propensity score matching based on demographic factors, yielding two balanced cohorts of 6638 patients each. The primary assessed outcomes over the five-year period post the diagnosis of stage 3 CKD included all-cause mortality (ICD-10: R99), major adverse cardiovascular events (MACE; ICD-10: I20-I25, I21, I46, I49, I50, I61, I63, R99), acute kidney injury (ICD-10: N17), pneumonia (ICD-10: J18.9), progression to GFR ≤ 30 mL/min (TNX: 8001), and GI bleeding (ICD-10: K92, K92.0, K25.0, K26.0, K29.01, K31.811, K92.1, K62.5, K57.33, I85.01, K92.2). Venous thromboembolism (VTE) was assessed as a secondary outcome. This structured approach ensures a rigorous comparison of clinical outcomes between iron-treated and untreated CKD stage 3 females with low serum ferritin but normal hemoglobin and MCV values.

### 2.3. Data Analysis

The data analysis for this study involved evaluating clinical outcomes in propensity-matched cohorts of stage 3 CKD, iron-deficient, non-anemic women with and without iron therapy over a follow-up period of five years. The primary outcomes assessed were all-cause mortality, MACE, AKI, pneumonia, GFR ≤ 30, and GI bleeding. Bonferroni correction across the six primary outcomes was performed to address the issue of multiple comparisons (corrected α = 0.0083). The presence of additional comorbidities such as diabetes mellitus, hypertension, obesity, ischemic heart disease, and cerebrovascular disease was also compared between the two cohorts.

Kaplan–Meier survival analysis was performed to calculate survival probabilities, and Cox proportional hazards regression models were applied to determine hazard ratios for all outcomes. Kaplan–Meier curves illustrate survival probability over time following an index event. The Kaplan–Meier analysis function in TriNetX censors patients if their last recorded clinical fact occurs within the observation period, leading to their exclusion from the Kaplan–Meier analysis. In contrast, TriNetX does not exclude these patients when computing cohort risks within a given time period. Therefore, we split the five-year observation period into separate 90-day intervals. We then determined the number of events and the corresponding risk for each 90-day interval (excluding repeated measures) and summed these values to obtain the total number of patients with the outcome over five years (90 × 21 = 1890 days). Using these manually aggregated data, cumulative survival rates were computed using the Kaplan–Meier estimator to compare cohorts using the log-rank test. The survival analysis was performed using the ‘survival’ R package for R software (version 4.4.2, Vienna, Austria).

Both *p*-values and corrected significance thresholds are reported to enhance transparency. This robust statistical framework ensured accurate and unbiased comparisons between the matched cohorts, providing a detailed understanding of the impact of iron therapy on clinical outcomes.

## 3. Results

### 3.1. Characteristics

Table 1 provides a detailed comparison of patient baseline characteristics before and after propensity score matching (PSM). Demographic data, comorbidity prevalence, medication usage, and laboratory values were compared between patients receiving iron therapy and patients who did not. Propensity score matching to eliminate differences in demographic variables was successful. Before matching, the iron therapy group (*n* = 6642) and the non-therapy group (*n* = 47,127) exhibited significant differences in race distribution (white and black or African American) at index (78.7% vs. 71.1% and 10.6% vs. 7.5%, respectively). After matching (*n* = 6638 per group), race at index was balanced.

Diagnosis prevalence varied significantly before matching, with higher rates of diabetes mellitus (10.0% vs. 6.0%, *p* < 0.001), overweight and obesity (7.3% vs. 4.8%, *p* < 0.001), ischemic heart disease (3.2% vs. 1.8%, *p* < 0.001), and cerebrovascular diseases (1.6% vs. 1.0%, *p* < 0.001) in the iron therapy group, and the difference became nonsignificant after PSM. Medication usage was also notably higher in the iron therapy group for antilipemic agents (12.1% vs. 7.4%, *p* < 0.001), beta blockers (9.3% vs. 5.5%, *p* < 0.001), calcium channel blockers (5.7% vs. 3.5%, *p* < 0.001), and angiotensin II inhibitors (5.1% vs. 3.2%, *p* < 0.001), with these differences diminishing post-matching. Laboratory values before matching showed significantly lower calcium (9.3 ± 0.7 vs. 9.4 ± 0.6 mg/dL, *p* < 0.001), lower albumin (4.0 ± 0.4 vs. 4.1 ± 0.4 g/dL, *p* < 0.001), lower total cholesterol (186.8 ± 47.6 vs. 193.6 ± 47.2 mg/dL, *p* = 0.001), and lower ferritin (40.2 ± 23.9 vs. 49.4 ± 24.5 ng/mL, *p* < 0.001) in the iron therapy group. The above disparities, including comorbidity, medication, and metabolic parameters, became nonsignificant after propensity score matching.

### 3.2. Five-Year Kaplan–Meier Curves to Evaluate Iron Therapy Primary Outcomes

**Figure 2:**
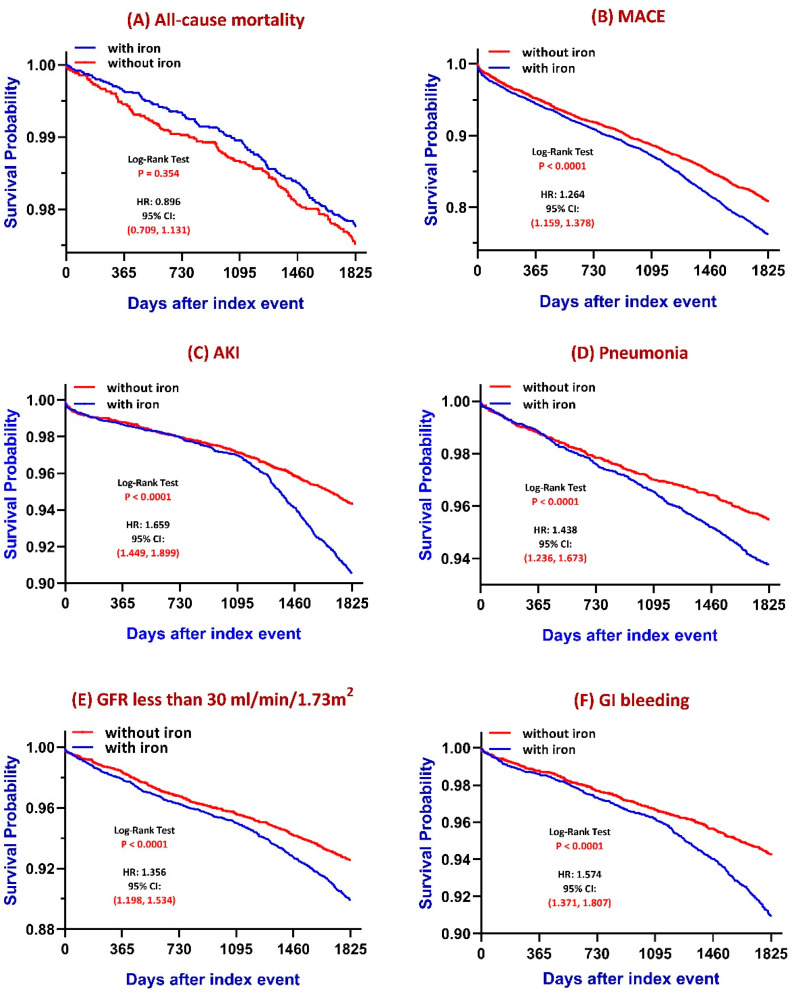
Five-year Kaplan–Meier curves to evaluate iron therapy outcomes.

The detailed outcome numbers were presented in Table S1. Kaplan–Meier survival analyses (Figure 2) demonstrated differential long-term risks between iron-treated and untreated patients. Over a 5-year follow-up:

1. All-cause mortality: No statistically significant difference was observed between groups (log-rank *p* = 0.354), although a subtle trend toward increased mortality was noted in the iron-treated group.
2. MACE: Patients receiving iron therapy had a significantly higher cumulative incidence of major adverse cardiovascular events compared to those without iron therapy (log-rank *p* < 0.0001).
3. AKI: The iron group exhibited a significantly greater risk of acute kidney injury (log-rank *p* < 0.0001). The difference is more prominent after long-term follow-up.
4. Pneumonia: Iron supplementation was associated with a significantly increased risk of pneumonia (log-rank *p* < 0.0001).
5. CKD progression: Defined as eGFR declining to ≤30 mL/min/1.73 m^2^, the iron-treated group showed a significantly faster decline in renal function (log-rank *p* < 0.0001).
6. GI bleeding: The increased risk of GI bleeding is observed in the iron-treated group (*p* < 0.0001). To specify the relationship between iron supplementation and GI bleeding, we confirmed the consistent outcomes both before and after matching (Table S2). Also, even excluding GI bleeding, these significant differences between the iron-treated group and the group without iron treatment were also unchanged (Table S3).

### 3.3. Secondary Outcomes: Venous Thromboembolism

In our analysis, iron supplementation is associated with an increased risk of venous thromboembolism (log-rank *p* < 0.0001, hazard ratio, 1.663, 95% CI, 1.323 to 2.092).

### 3.4. Subgroup Analysis of Iron Therapy

**Figure 3:**
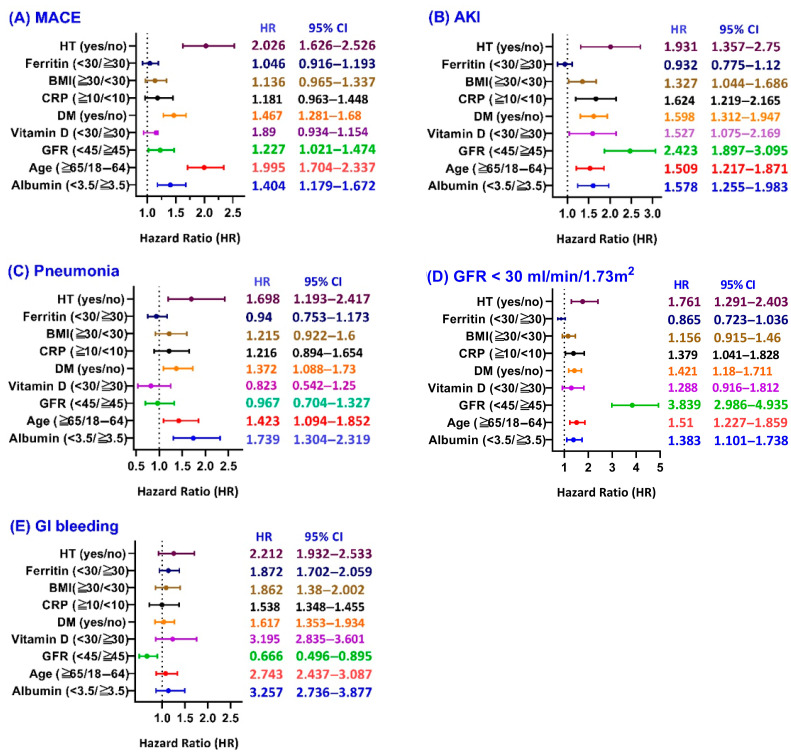
Subgroup analysis of iron therapy vs. no iron therapy.

In subgroup analyses, iron therapy was associated with increased risks of adverse outcomes in selected patient groups. For MACE in Figure 3A, the association was statistically significant in patients with hypertension (hazard ratio, 2.026, 95% confidence interval [CI], 1.626 to 2.526), those aged ≥65 years (hazard ratio, 1.995, 95% CI, 1.704 to 2.337), patients with GFR < 45 mL/min/1.73 m^2^ (hazard ratio, 1.227, 95% CI, 1.021 to 1.474), those with diabetes mellitus (hazard ratio, 1.467, 95% CI, 1.281 to 1.680), and those with low serum albumin (hazard ratio, 1.404, 95% CI, 1.179 to 1.672). For AKI in Figure 3B, iron therapy was significantly associated with a higher risk in all subgroups except patients with serum ferritin < 30 (ng/mL). Increased risk of pneumonia was evident in the subgroup with hypertension (hazard ratio, 1.689, 95% CI, 1.193 to 2.417), diabetes mellitus (hazard ratio 1.372, 95% CI, 1.088 to 1.730), aged ≥ 65 years (hazard ratio 1.423, 95% CI, 1.094 to 1.852), and serum albumin < 3.5 g/dL (hazard ratio 1.739, 95% CI, 1.101 to 1.738). Other subgroups did not show statistically significant differences (Figure 3C). Iron therapy was significantly associated with CKD progression (defined as eGFR ≤ 30 mL/min/1.73 m^2^) in patients aged ≥ 65 years (hazard ratio, 1.761, 95% CI, 1.291 to 2.403), those with CRP ≥10 mg/L (hazard ratio 1.379, 95% CI, 1.041 to 1.828), diabetes mellitus (hazard ratio 1.421, 95% CI, 1.180 to 1.711), GFR <45 mL/min/1.73 m^2^ (hazard ratio 3.839, 95% CI, 2.986 to 4.935), aged ≥ 65 years (hazard ratio 1.51, 95% CI, 1.277 to 1.859), and serum albumin < 3.5 g/dL (hazard ratio 1.383, 95% CI, 1.101 to 1.738) (Figure 3D). In Figure 3E, only GFR < 45 mL/min/1.73 m^2^ has a decreased risk of GI bleeding (hazard ratio 0.666, 95% CI, 0.496 to 0.895). Other subgroups showed no statistically significant differences.

## 4. Discussion

Our study demonstrated that in non-anemic, iron-deficient women with stage 3 CKD and low ferritin levels, the incidence of major adverse cardiovascular events (MACE), pneumonia, AKI, progression to advanced kidney diseases (eGFR < 30 mL/min/1.73 m^2^), and GI bleeding were all significantly higher in patients receiving iron supplementation therapy.

The benefits of iron supplementation in non-anemic CKD patients with low ferritin remain uncertain. Some studies have investigated the effects of iron therapy in non-anemic individuals with low ferritin, including a randomized controlled trial (RCT) which found intravenous iron administration significantly reduced fatigue levels in premenopausal women with ferritin ≤ 15 ng/mL [19,20]. The 2025 KDIGO guidelines acknowledge the potential clinical benefit of iron supplementation in CKD patients with profound iron deficiency, based on specific ferritin and transferrin saturation thresholds [13,21,22]. However, routine iron supplementation in non-anemic CKD patients with low ferritin is not universally recommended due to the lack of clear benefits and potential health risks. The decision to initiate iron supplementation in this patient cohort therefore lacks supporting evidence and remains a difficult decision according to individualized conditions. Our findings address this evidence gap by providing real-world outcome data from a large, propensity-matched cohort of non-anemic women with CKD stage 3 and low serum ferritin. This retrospective analysis demonstrates how iron supplementation potentially influences renal function and the risk of MACE, pneumonia, and GI bleeding in IDWA women. Our findings provide timely and complementary support to assist clinical decision-making, particularly in the context of guideline ambiguity.

### 4.1. Mortality and MACE

A prospective analysis also found that absolute iron deficiency (serum ferritin < 30 µg/mL) in women was associated with a 1.77-fold increased mortality risk [23]. A recent multicenter retrospective cohort study even demonstrated that non-dialysis-dependent CKD patients without anemia but with transferrin saturation (TSAT) levels ≤ 20% experienced a 2.21-fold increase in all-cause mortality risk (adjusted hazard ratio [aHR]: 2.21; 95% confidence interval [CI]: 1.36–3.57; *p* = 0.001) and a 1.66-fold increase in CKD progression compared to those with TSAT levels >20% [9]. However, their results conflict with other studies which found iron supplementation was associated with reduced mortality [11,18,24]. The possible explanation for the conflicting condition is the non-linear relationship between iron status and outcomes, which indicates the necessity of close follow-up to avoid iron overload. Prior research suggested a J-shaped association between iron dose and mortality risk, with the lowest hazard observed at approximately 12 mg per day of elemental iron [25,26,27]. Our study found no significant difference in all-cause mortality between the iron and non-iron groups. Moreover, we found that iron therapy was associated with a significantly higher risk of major adverse cardiovascular events (MACE) over a 5-year follow-up. Both iron deficiency and iron overload may be harmful, particularly in susceptible populations. From a mechanistic perspective, excessive or inappropriate iron administration can be associated with increased oxidative stress, endothelial dysfunction, and inflammatory activation, which are all established contributors to atherosclerosis and cardiovascular complications. In patients with CKD, especially those with concurrent heart failure, dysregulated iron metabolism and systemic inflammation may further exacerbate cardiovascular risk. Although iron may improve functional capacity in CHF, the absence of anemia in our cohort may limit the therapeutic benefit, while the potential for iron-induced harm remains. Taken together, our findings highlight the need for precision in iron therapy, particularly in non-anemic CKD women. Iron supplementation should not be broadly applied without considering iron indices, cardiovascular risk, comorbid conditions, and optimal dosing thresholds.

### 4.2. AKI and CKD Progression

The increased risk of AKI and CKD progression is consistent with previous studies [28,29]. Iron overload catalyzes the Fenton reaction, leading to the generation of reactive oxygen species, lipid peroxidation, and oxidative damage to tubular epithelial cells. Inflammatory signaling further exacerbates iron retention by upregulating iron transporters such as DMT1 and Zip14, while hepcidin-induced ferroportin suppression impairs cellular iron export. This dysregulated iron handling contributes to cellular injury and interstitial fibrosis. Ferroptosis, a distinct form of iron-dependent programmed cell death, has also been implicated in the pathophysiology of AKI. It is triggered by multiple mechanisms including impairment of system Xc^−^ (cystine/glutamate antiporter), glutathione peroxidase 4 (GPX4) inhibition, iron overload, ROS generation, and lipid peroxidation [30,31]. These cellular events are associated with necroinflammation, autophagy dysfunction, and structural renal damage, ultimately contributing to the progression of kidney injury.

### 4.3. Pneumonia

In female patients with stage 3 CKD, maintaining adequate iron stores is critical for optimal immune function, as iron supports the activity of neutrophils, lymphocytes, and other immune cells [32]. However, iron supplementation has been reported to increase the risk of infection, pneumonia, and the length of hospital days [33,34]. Iron supplementation could be associated with microbiota-immune responses by shifting gut microbiota to a different profile [35]. Both oral and intravenous iron supplementation may therefore be associated with various infection risks, which must be considered alongside the potential benefit of correcting absolute iron deficiency.

### 4.4. Gastrointestinal Bleeding

Gastrointestinal bleeding is more common in chronic kidney disease, especially in advanced stages. Key predictors include older age, diabetes, peptic ulcer disease, cirrhosis, and angiodysplasia, which causes 30–44% of cases [36]. Oral iron supplementation is associated with gastrointestinal side effects [37]. In our cohort, iron therapy is associated with increasing bleeding risk both before and after matching. These findings remind us of cautious iron use and close monitoring in this subgroup of patients.

### 4.5. Venous Thromboembolism

Given the predisposition to VTE in iron deficiency patients, we included VTE as a secondary outcome [38]. Notably, our findings suggest that iron supplementation is associated with an increased risk of VTE. Iron-induced oxidative stress has been implicated as a potential risk factor [39]. A more precise dose–response study may be warranted to clarify this issue.

A major strength of our study is the use of a large, real-world multicenter database, allowing us to examine clinically relevant outcomes in a well-defined niche population of non-anemic, iron-deficient women with stage 3 CKD. Our study specifically focused on a subgroup that is underrepresented in prior trials and observational studies, providing novel insight into the risks associated with iron therapy in this population. However, our study has several limitations. First, the electronic health record coding accuracy is a known inevitable limitation. Second, as a retrospective cohort analysis, causal relationships cannot be confirmed. Due to the unavailability of TSAT values, ferritin < 100 ng/mL was used as a surrogate marker of iron status. Both groups exhibited elevated CRP levels, indicative of systemic inflammation. Given that ferritin is an acute-phase reactant, this may have resulted in the misclassification of functional iron deficiency as absolute iron deficiency. Third, to ensure adequate case numbers, we adopted a broad definition of iron therapy. Data on functional outcomes and quality of life were not captured in the database. Additionally, the form, dose, and duration of iron supplementation were not recorded, posing a residual indication bias and preventing us from performing a dose–response evaluation. Lastly, patients receiving iron may have been in a more compromised state of health, resulting in potential confounding by indication.

An additional limitation brought to our attention during peer review is that we did not balance medication confounders known to influence gastrointestinal bleeding and iron loss. We therefore reperformed our main analysis with the use of the following medications balanced during propensity score matching: non-steroidal anti-inflammatory drugs, aspirin, rivaroxaban, edoxaban, and dabigatran. The outcomes of this supplementary analysis are nearly identical (Table S4), demonstrating that the confounding effect of medication use has likely not influenced the results of the above study.

## 5. Conclusions

Iron deficiency in CKD patients is a concern. The specific mortality risk for non-anemic female patients with stage 3 CKD and low serum ferritin is not well-defined. The potential benefits of iron therapy in this subgroup require further investigation to establish clear guidelines. In the absence of anemia, iron therapy may not confer cardiovascular protection and could potentially exacerbate underlying vascular or inflammatory processes, leading to higher MACE risk. It is important to note that excessive iron carries potential risks, including iron overload and increased oxidative stress, which may adversely affect renal function. Therefore, the decision to initiate iron therapy in a non-anemic CKD female with low ferritin should be individualized, carefully weighing potential benefits against risks.

## Figures and Tables

**Table 1:** Patient baseline characteristics before and after propensity score matching.

	Before Matching				After Matching			
	With Iron (*n* = 6642)	Without Iron (*n* = 47,127)	*p* Value	Std Diff	With Iron (*n* = 6638)	Without Iron (*n* = 6638)	*p* Value	Std Diff
General data								
Age	59.4 ± 13.8	59.1 ± 12.8	0.106	0.021	59.4 ± 13.8	59.6 ± 13.2	0.297	0.018
White	78.7%	71.1%	<0.001	0.177	78.7%	79.7%	0.165	0.024
Black or African American	10.6%	7.5%	<0.001	0.107	10.6%	10.8%	0.633	0.008
Asian	1.2%	1.2%	0.928	0.001	1.2%	1.2%	0.690	0.007
Comorbidity								
Diabetes mellitus	10.0%	6.0%	<0.001	0.149	9.9%	9.7%	0.705	0.007
Hypertensive diseases	21.4%	21.3%	0.918	0.004	20.9%	20.0%	0.196	0.022
Overweight and obesity	7.3%	4.8%	<0.001	0.104	7.3%	7.4%	0.868	0.003
Ischemic heart disease	3.2%	1.8%	<0.001	0.087	3.2%	2.9%	0.364	0.016
Cerebrovascular diseases	1.6%	1.0%	<0.001	0.051	1.6%	1.4%	0.518	0.011
Medications								
Antilipemic agents	12.1%	7.4%	<0.001	0.156	12.0%	11.4%	0.268	0.019
Beta blockers	9.3%	5.5%	<0.001	0.146	9.3%	8.4%	0.076	0.031
Calcium channel blockers	5.7%	3.5%	<0.001	0.104	5.6%	5.5%	0.622	0.009
Angiotensin II inhibitors	5.1%	3.2%	<0.001	0.099	5.1%	4.5%	0.115	0.027
NSAIDs	2.9%	1.9%	<0.001	0.064	2.9%	3.0%	0.642	0.009
Aspirin	4.8%	2.5%	<0.001	0.127	4.7%	4.0%	0.045	0.035
Rivaroxban	0.5%	0.3%	0.002	0.036	0.5%	0.4%	0.361	0.016
Edoxaban	0.2%	0.02%	<0.001	0.044	0.2%	0%	0.002	0.055
Dabigatran	0.2%	0.1%	0.003	0.032	0.2%	0.2%	1	<0.001
Laboratory exams								
Creatinine, mg/dL	0.9 ± 0.3	0.9 ± 0.9	0.346	0.034	0.9 ± 0.3	1.0 ± 1.3	0.153	0.054
Calcium, mg/dL	9.3 ± 0.7	9.4 ± 0.6	<0.001	0.106	9.3 ± 0.7	9.4 ± 0.6	0.166	0.056
Phosphate, mg/dL	3.6 ± 0.7	3.6 ± 0.7	0.990	0.001	3.6 ± 0.7	3.6 ± 0.8	0.566	0.061
Hemoglobin, g/dL	13.8 ± 1.1	13.8 ± 1.0	0.361	0.030	13.8 ± 1.1	13.8 ± 1.1	0.478	0.032
MCV, fL	90.6 ± 4.6	90.7 ± 4.5	0.276	0.037	90.6 ± 4.6	90.6 ± 4.7	0.793	0.012
Alk phosphatase, U/L	82.5 ± 31.1	82.7 ± 39.8	0.907	0.005	82.5 ± 31.1	83.6 ± 32.7	0.482	0.034
Albumin, g/dL	4.0 ± 0.4	4.1 ± 0.4	<0.001	0.216	4.0 ± 0.4	4.0 ± 0.5	0.088	0.081
Total cholesterol, mg/dL	186.8 ± 47.6	193.6 ± 47.2	0.001	0.144	186.9 ± 47.7	190.2 ± 53.4	0.282	0.065
Hemoglobin A1c, %	6.4 ± 1.3	6.4 ± 1.4	0.980	0.002	6.9 ± 1.8	6.8 ± 1.7	0.457	0.048
Intact PTH, pg/mL	62.3 ± 44.9	62.5 ± 43.0	0.980	0.003	62.3 ± 44.9	68.4 ± 51.9	0.508	0.125
Iron, ug/dL	80.1 ± 38.2	83.1 ± 36.2	0.330	0.082	80.1 ± 38.2	85.2 ± 42.1	0.282	0.127
Ferritin, ng/mL	40.2 ± 23.9	49.4 ± 24.5	<0.001	0.380	40.2 ± 23.9	51.0 ± 25.6	0.409	0.014
CRP, mg/L	11.8 ± 18.7	8.8 ± 20.3	0.097	0.153	11.9 ± 18.8	12.7 ± 30.3	0.778	0.034

## Data Availability

All data were extracted from the TriNetX network. Further enquiries can be directed to the corresponding author.

## Supplementary Materials

The following supporting information can be downloaded at https://www.mdpi.com/article/10.3390/jcm14155575/s1, Table S1. Five-year risk analysis of outcomes in non-anemic female patients with stage 3 chronic kidney disease, comparing those who received iron therapy versus those who did not. Table S2. Five-year Kaplan–Meier survival curve analysis of outcomes in non-anemic female patients with stage 3 chronic kidney disease, comparing hazard ratios between the unmatched cohort (before matching) and the matched cohort (after matching) derived from the entire patient population. Table S3. Five-year Kaplan–Meier survival analysis of outcomes in non-anemic female patients with stage 3 chronic kidney disease, comparing hazard ratios between the matched cohort excluding gastrointestinal bleeding and the matched cohort without this exclusion. Table S4. Re-analysis of main cohort outcomes with additional medication confounders balanced during propensity score matching.

## Abbreviations

The following abbreviations are used in this manuscript:

IDWA	Iron deficiency without anemia
TSAT	Transferrin saturation
