# Supplementary material for: Clinical Outcomes of Iron Supplement Therapy in Non-Anemic Female CKD Stage 3 Patients with Low Serum Ferritin Level: A Multi-Institutional TriNetX Analysis
Source: J Clin Med. 2025 Aug 7;14(15):5575. doi: 10.3390/jcm14155575 (PMC12347412; doi:10.3390/jcm14155575)
Supplement: Supplementary file 1 [file jcm-14-05575-s001.zip › Supplement Table S1.pptx]

## Slide 1
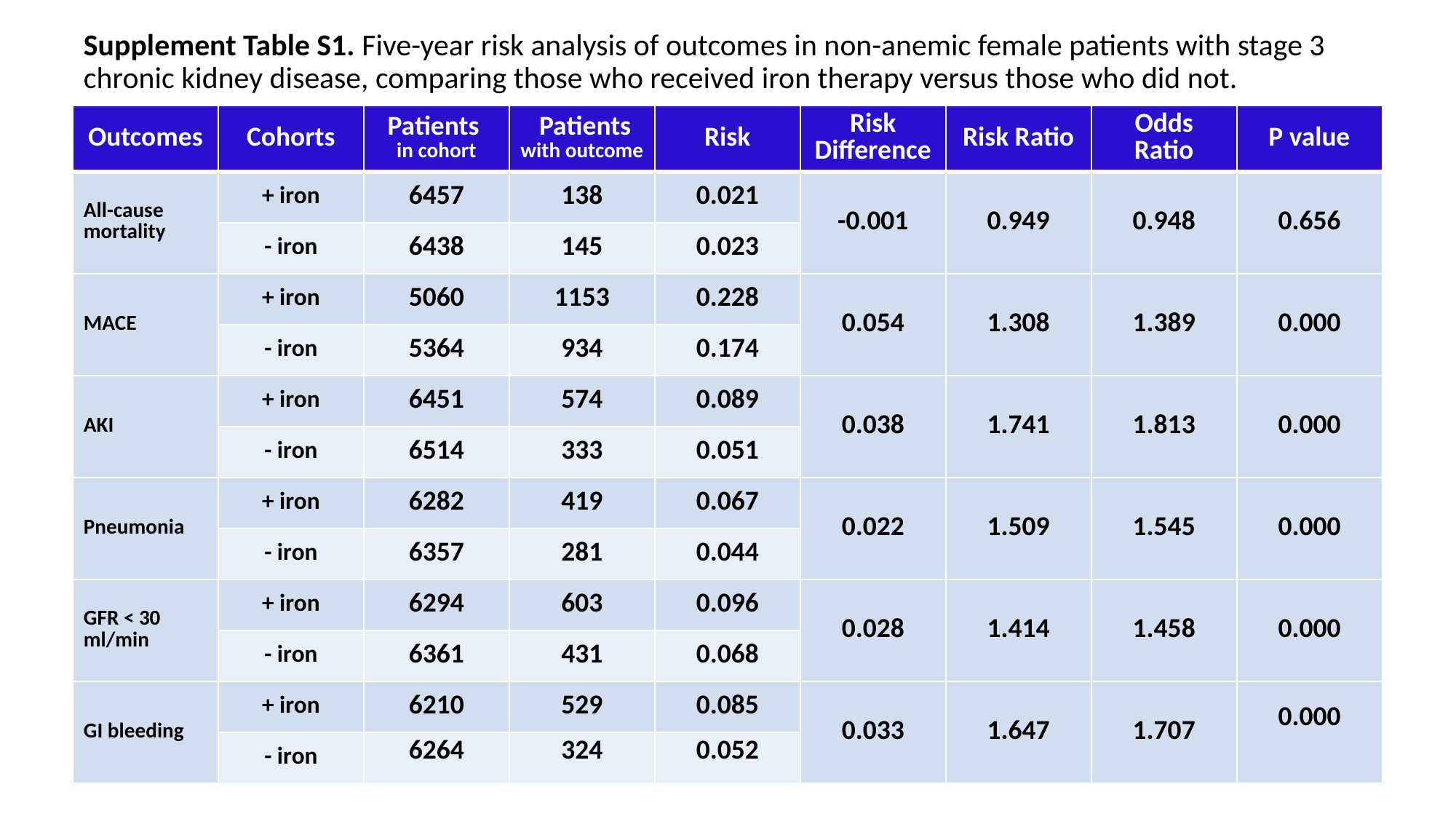

# Supplement Table S1. Five-year risk analysis of outcomes in non-anemic female patients with stage 3 chronic kidney disease, comparing those who received iron therapy versus those who did not.
| Outcomes | Cohorts | Patients in cohort | Patients with outcome | Risk | Risk Difference | Risk Ratio | Odds Ratio | P value |
| --- | --- | --- | --- | --- | --- | --- | --- | --- |
| All-cause mortality | + iron | 6457 | 138 | 0.021 | -0.001 | 0.949 | 0.948 | 0.656 |
| | - iron | 6438 | 145 | 0.023 | | | | |
| MACE | + iron | 5060 | 1153 | 0.228 | 0.054 | 1.308 | 1.389 | 0.000 |
| | - iron | 5364 | 934 | 0.174 | | | | |
| AKI | + iron | 6451 | 574 | 0.089 | 0.038 | 1.741 | 1.813 | 0.000 |
| | - iron | 6514 | 333 | 0.051 | | | | |
| Pneumonia | + iron | 6282 | 419 | 0.067 | 0.022 | 1.509 | 1.545 | 0.000 |
| | - iron | 6357 | 281 | 0.044 | | | | |
| GFR < 30 ml/min | + iron | 6294 | 603 | 0.096 | 0.028 | 1.414 | 1.458 | 0.000 |
| | - iron | 6361 | 431 | 0.068 | | | | |
| GI bleeding | + iron | 6210 | 529 | 0.085 | 0.033 | 1.647 | 1.707 | 0.000 |
| | - iron | 6264 | 324 | 0.052 | | | | |
