# Supplementary material for: Clinical Outcomes of Iron Supplement Therapy in Non-Anemic Female CKD Stage 3 Patients with Low Serum Ferritin Level: A Multi-Institutional TriNetX Analysis
Source: J Clin Med. 2025 Aug 7;14(15):5575. doi: 10.3390/jcm14155575 (PMC12347412; doi:10.3390/jcm14155575)
Supplement: Supplementary file 1 [file jcm-14-05575-s001.zip › Supplement Table S2.pptx]

## Slide 1
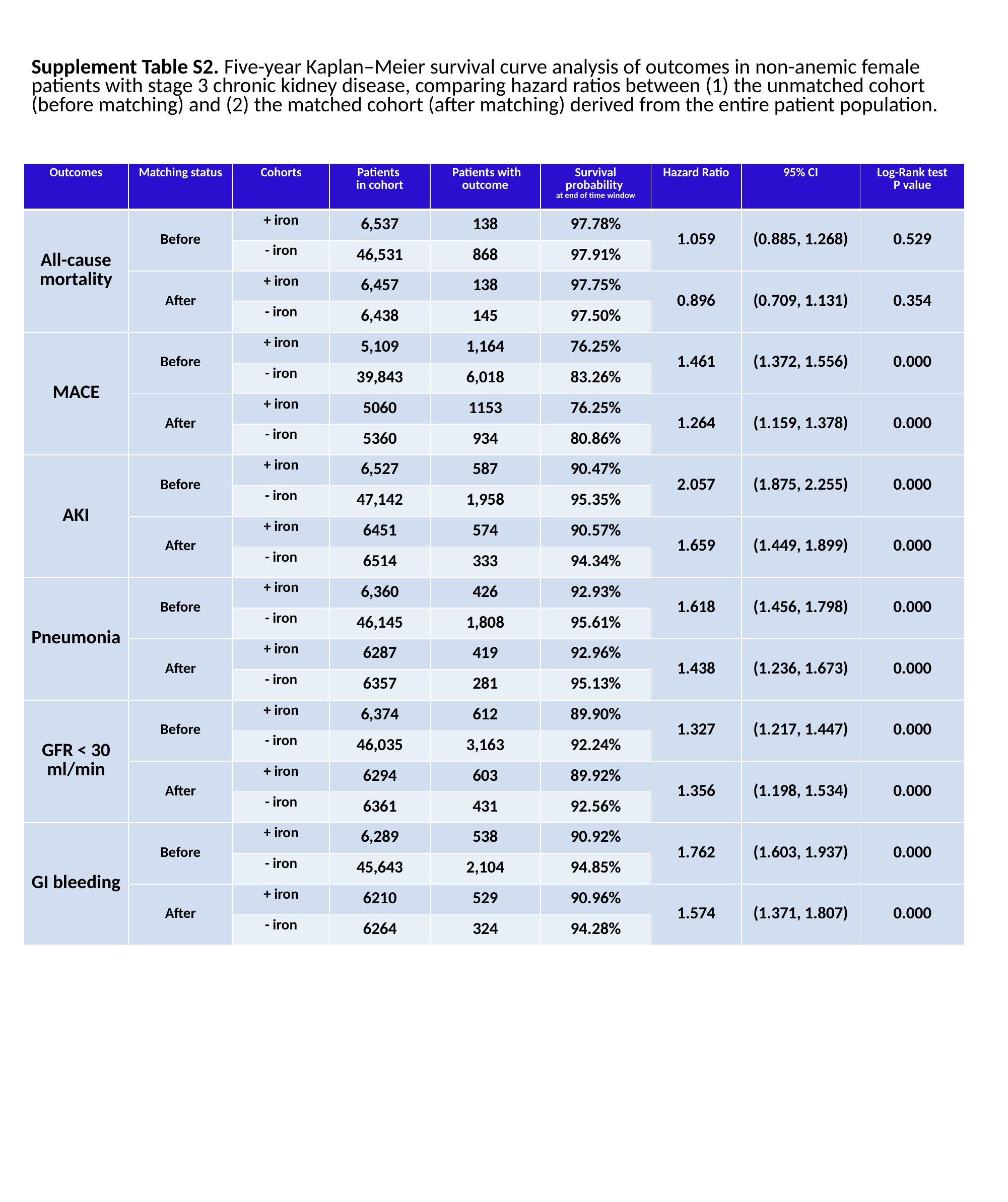

# Supplement Table S2. Five-year Kaplan–Meier survival curve analysis of outcomes in non-anemic female patients with stage 3 chronic kidney disease, comparing hazard ratios between (1) the unmatched cohort (before matching) and (2) the matched cohort (after matching) derived from the entire patient population.
| Outcomes | Matching status | Cohorts | Patients in cohort | Patients with outcome | Survival probability at end of time window | Hazard Ratio | 95% CI | Log-Rank test P value |
| --- | --- | --- | --- | --- | --- | --- | --- | --- |
| All-cause mortality | Before | + iron | 6,537 | 138 | 97.78% | 1.059 | (0.885, 1.268) | 0.529 |
| | | - iron | 46,531 | 868 | 97.91% | | | |
| | After | + iron | 6,457 | 138 | 97.75% | 0.896 | (0.709, 1.131) | 0.354 |
| | | - iron | 6,438 | 145 | 97.50% | | | |
| MACE | Before | + iron | 5,109 | 1,164 | 76.25% | 1.461 | (1.372, 1.556) | 0.000 |
| | | - iron | 39,843 | 6,018 | 83.26% | | | |
| | After | + iron | 5060 | 1153 | 76.25% | 1.264 | (1.159, 1.378) | 0.000 |
| | | - iron | 5360 | 934 | 80.86% | | | |
| AKI | Before | + iron | 6,527 | 587 | 90.47% | 2.057 | (1.875, 2.255) | 0.000 |
| | | - iron | 47,142 | 1,958 | 95.35% | | | |
| | After | + iron | 6451 | 574 | 90.57% | 1.659 | (1.449, 1.899) | 0.000 |
| | | - iron | 6514 | 333 | 94.34% | | | |
| Pneumonia | Before | + iron | 6,360 | 426 | 92.93% | 1.618 | (1.456, 1.798) | 0.000 |
| | | - iron | 46,145 | 1,808 | 95.61% | | | |
| | After | + iron | 6287 | 419 | 92.96% | 1.438 | (1.236, 1.673) | 0.000 |
| | | - iron | 6357 | 281 | 95.13% | | | |
| GFR < 30 ml/min | Before | + iron | 6,374 | 612 | 89.90% | 1.327 | (1.217, 1.447) | 0.000 |
| | | - iron | 46,035 | 3,163 | 92.24% | | | |
| | After | + iron | 6294 | 603 | 89.92% | 1.356 | (1.198, 1.534) | 0.000 |
| | | - iron | 6361 | 431 | 92.56% | | | |
| GI bleeding | Before | + iron | 6,289 | 538 | 90.92% | 1.762 | (1.603, 1.937) | 0.000 |
| | | - iron | 45,643 | 2,104 | 94.85% | | | |
| | After | + iron | 6210 | 529 | 90.96% | 1.574 | (1.371, 1.807) | 0.000 |
| | | - iron | 6264 | 324 | 94.28% | | | |
