# Supplementary material for: Clinical Outcomes of Iron Supplement Therapy in Non-Anemic Female CKD Stage 3 Patients with Low Serum Ferritin Level: A Multi-Institutional TriNetX Analysis
Source: J Clin Med. 2025 Aug 7;14(15):5575. doi: 10.3390/jcm14155575 (PMC12347412; doi:10.3390/jcm14155575)
Supplement: Supplementary file 1 [file jcm-14-05575-s001.zip › Supplement Table S3.pptx]

## Slide 1
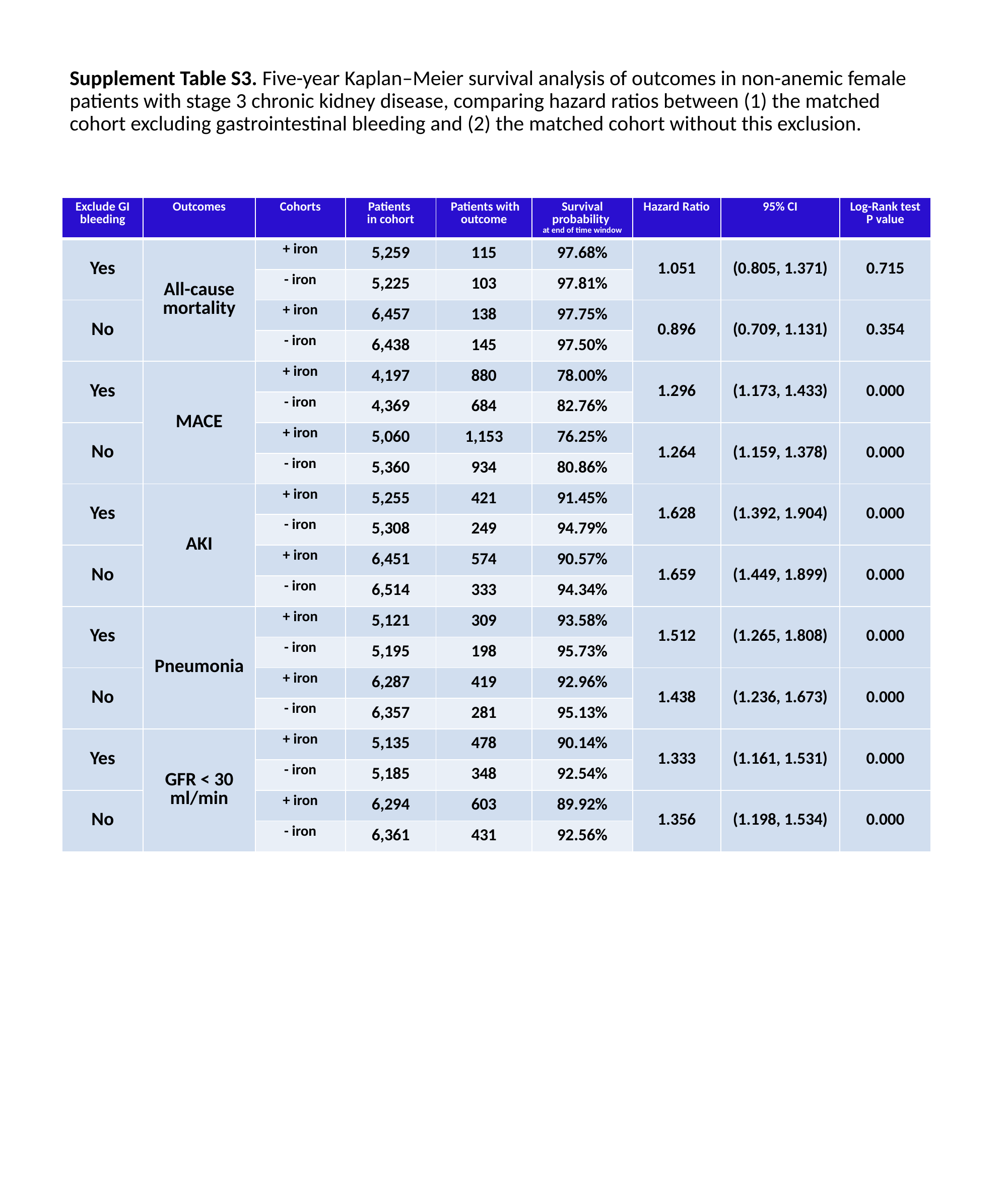

# Supplement Table S3. Five-year Kaplan–Meier survival analysis of outcomes in non-anemic female patients with stage 3 chronic kidney disease, comparing hazard ratios between (1) the matched cohort excluding gastrointestinal bleeding and (2) the matched cohort without this exclusion.
| Exclude GI bleeding | Outcomes | Cohorts | Patients in cohort | Patients with outcome | Survival probability at end of time window | Hazard Ratio | 95% CI | Log-Rank test P value |
| --- | --- | --- | --- | --- | --- | --- | --- | --- |
| Yes | All-cause mortality | + iron | 5,259 | 115 | 97.68% | 1.051 | (0.805, 1.371) | 0.715 |
| | | - iron | 5,225 | 103 | 97.81% | | | |
| No | | + iron | 6,457 | 138 | 97.75% | 0.896 | (0.709, 1.131) | 0.354 |
| | | - iron | 6,438 | 145 | 97.50% | | | |
| Yes | MACE | + iron | 4,197 | 880 | 78.00% | 1.296 | (1.173, 1.433) | 0.000 |
| | | - iron | 4,369 | 684 | 82.76% | | | |
| No | | + iron | 5,060 | 1,153 | 76.25% | 1.264 | (1.159, 1.378) | 0.000 |
| | | - iron | 5,360 | 934 | 80.86% | | | |
| Yes | AKI | + iron | 5,255 | 421 | 91.45% | 1.628 | (1.392, 1.904) | 0.000 |
| | | - iron | 5,308 | 249 | 94.79% | | | |
| No | | + iron | 6,451 | 574 | 90.57% | 1.659 | (1.449, 1.899) | 0.000 |
| | | - iron | 6,514 | 333 | 94.34% | | | |
| Yes | Pneumonia | + iron | 5,121 | 309 | 93.58% | 1.512 | (1.265, 1.808) | 0.000 |
| | | - iron | 5,195 | 198 | 95.73% | | | |
| No | | + iron | 6,287 | 419 | 92.96% | 1.438 | (1.236, 1.673) | 0.000 |
| | | - iron | 6,357 | 281 | 95.13% | | | |
| Yes | GFR < 30 ml/min | + iron | 5,135 | 478 | 90.14% | 1.333 | (1.161, 1.531) | 0.000 |
| | | - iron | 5,185 | 348 | 92.54% | | | |
| No | | + iron | 6,294 | 603 | 89.92% | 1.356 | (1.198, 1.534) | 0.000 |
| | | - iron | 6,361 | 431 | 92.56% | | | |
