# Supplementary material for: Clinical Outcomes of Iron Supplement Therapy in Non-Anemic Female CKD Stage 3 Patients with Low Serum Ferritin Level: A Multi-Institutional TriNetX Analysis
Source: J Clin Med. 2025 Aug 7;14(15):5575. doi: 10.3390/jcm14155575 (PMC12347412; doi:10.3390/jcm14155575)
Supplement: Supplementary file 1 [file jcm-14-05575-s001.zip › Supplement Table S4.pdf]

Table S4: Re-analysis of main cohort outcomes with additional medication confounders balanced during propensity score matching

Compare cohort	Outcome	Aspirin & anticoagulants	Hazard Ratio	95 % CI	Log-Rank Test p
F<100 with iron vs. F<100 without iron	All-cause mortality	Not balanced	0.896	0.709—1.131	0.354
		Balanced	0.870	0.689—1.098	0.241
F<100 with iron vs. F<100 without iron	MACE	Not balanced	1.264	1.159—1.378	<0.0001
		Balanced	1.295	1.191—1.407	<0.0001
F<100 with iron vs. F<100 without iron	AKI	Not balanced	1.659	1.449—1.899	<0.0001
		Balanced	1.587	1.398—1.801	<0.0001
F<100 with iron vs. F<100 without iron	Pneumonia	Not balanced	1.438	1.236—1.673	<0.0001
		Balanced	1.530	1.321—1.772	<0.0001
F<100 with iron vs. F<100 without iron	GFR<30 ml/min/1.73m^2	Not balanced	1.356	1.198—1.534	<0.0001
		Balanced	1.359	1.200—1.539	<0.0001
F<100 with iron vs. F<100 without iron	GI bleeding	Not balanced	1.574	1.371—1.807	<0.0001
		Balanced	1.582	1.385—1.806	<0.0001
